# Elevated exhaled acetone concentration in stage C heart failure patients with diabetes mellitus

**DOI:** 10.1186/s12872-017-0713-0

**Published:** 2017-11-16

**Authors:** Tetsuro Yokokawa, Takamasa Sato, Satoshi Suzuki, Masayoshi Oikawa, Akiomi Yoshihisa, Atsushi Kobayashi, Takayoshi Yamaki, Hiroyuki Kunii, Kazuhiko Nakazato, Hitoshi Suzuki, Shu-ichi Saitoh, Takafumi Ishida, Akito Shimouchi, Yasuchika Takeishi

**Affiliations:** 1 Department of Cardiovascular Medicine, Fukushima Medical University, 1 Hikarigaoka, Fukushima, Fukushima 960-1295 Japan; 2 Department of Lifelong Sports for Health Biochemical Sciences, College of Life and Health Sciences, Chubu University, Kasugai, Aichi Japan

**Keywords:** Acetone, Breath analysis, Heart failure, Diabetes mellitus

## Abstract

**Background:**

Breath acetone is a noninvasive biomarker of heart failure; however, its significance in heart failure patients with diabetes mellitus has yet to be clarified. The objective of this study is to investigate whether exhaled acetone concentration is a noninvasive biomarker in heart failure patients with diabetes mellitus.

**Methods:**

This study prospectively included 35 diabetic patients with stage C heart failure and 20 diabetic patients with or at risk of heart failure (stage A or B). Exhaled breath was collected after an overnight fast.

**Results:**

The stage C group had significantly higher brain natriuretic peptide levels, larger left ventricular diameter, lower left ventricular ejection fraction, and more frequent use of β-blocker, compared with the stage A or B group. The stage C group had higher exhaled acetone concentrations than the stage A or B group (*p* = 0.013). Exhaled acetone concentration was correlated with total ketone bodies (*r* = 0.588, *p* < 0.001) and brain natriuretic peptide (*r* = 0.415, *p* = 0.002).

**Conclusion:**

Stage C heart failure patients with diabetes mellitus have elevated exhaled acetone concentrations. Exhaled acetone concentration could be a noninvasive biomarker in heart failure patients with diabetes mellitus.

## Background

Energy metabolism mainly depends on fatty acids and glucose in the heart [1]. However, the failing heart relies on ketone bodies, which are minor substrates for the normal myocardium [2, 3]. In addition, ketone body utilization is impaired in the skeletal muscle, as systemic deconditioning in heart failure [4]. Elevated myocardial energy expenditure is associated with significant changes of 3-hydroxybutyrate and acetone, which are ketone bodies [5]. These altered cardiac and systemic metabolism causes elevated blood ketone body levels in heart failure patients [6].

Acetone, a volatile component of ketone bodies, is detected in the breath. Breath acetone concentration is elevated in heart failure, following the elevation of blood ketone body levels, and has been reported to be a noninvasive biomarker of heart failure [7, 8]. Exhaled acetone might also be detected in patients with diabetes mellitus, lung cancer, and allergic asthma [9–11].

Among the diseases related to exhaled acetone, diabetes mellitus is a representative cause of ketosis, and is an important comorbidity in heart failure patients [12, 13]. As far as we know, only one report described exhaled acetone in a heterogeneous group with heart failure, including some diabetes mellitus patients [14]. Exhaled acetone concentration has not been investigated in diabetic patients with heart failure only. We investigated whether exhaled acetone concentration could be a noninvasive biomarker in heart failure patients with diabetes mellitus.

## Methods

This cross sectional study included 35 patients with stage C heart failure and diabetes mellitus (stage C group) who were hospitalized for 6 months between September 2016 and March 2017 in Fukushima Medical University Hospital [15]. Diagnosis of heart failure was evaluated by two or more independent cardiologists with two major criteria or one major criterion in conjunction with two minor Framingham criteria [16]. Heart failure was categorized in subtypes A, B and C according to the American College of Cardiology Foundation/American Heart Association guidelines [15]. Stages A and B patients were defined as those with risk factors that clearly predispose toward the development of heart failure [15]. Stage C denoted patients with current or past symptoms of heart failure associated with underlying structural heart disease [15]. Diabetes mellitus was defined as the recent use of antidiabetic drugs, a fasting blood glucose value of ≥126 mg/dL and/or a hemoglobin A1c value of ≥6.5%. The exclusion criteria were patients with hyperglycemic crisis including diabetic ketoacidosis, lung cancer, and allergic asthma. Twenty patients with or at risk of heart failure (stage A or B heart failure) and diabetes mellitus were also enrolled (stage A or B group) [15]. The stage A or B group included 10 cases of angina pectoris, five cases of previous myocardial infarction, two cases of hypertension, two cases of atrial fibrillation, and one case of sick sinus syndrome. Baseline data of sex, age, body mass index, New York Heart Association class, etiology of heart failure, past medical history, physical findings, and current medication were collected at the time of enrollment in this study. This study protocol was approved by the Institutional Ethics Committee of Fukushima Medical University. Written informed consent was provided by all patients.

### Blood test and breath analysis

All patients received a hospital diet with a median intake (interquartile range) of total energy, carbohydrate, protein, and fat was 1600 (1600–1800) kcal/day, 240 (235–263) g, 70 (66–78) g, and 41 (41–48) g, respectively. Blood samples were analyzed for estimated glomerular filtration rate (eGFR), total cholesterol, total bilirubin, glucose, hemoglobin A1c, brain natriuretic peptide, and total ketone bodies. Breath was collected for analysis once in the early morning after at least a 12-h fast at a stable phase. A breath-sampling bag (Collection Bag, Laboratory for Expiration Biochemistry, Nourishment Metabolism Co., Ltd., Nara, Japan) was used to maintain the breath. The collected breath was transferred to a gas-tight glass syringe, and 5 ml was injected into a gas analysis device (GC-8A; Shimadzu Co., Ltd., Kyoto, Japan) to measure breath acetone concentration within the day of breath collection. Every measurement of the breath acetone was calibrated by standard gas (Sumitomo Seika Chemicals Co., LTD., Osaka, Japan). Acetone concentration in the breath of each patient was calculated by subtracting the acetone concentration of ambient air around the patients measured using the same method for the breath. The sampling method to evaluate exhaled acetone was previously reported and only the gas analysis device was different from the previous study [17].

### Echocardiography

Echocardiography was performed by a blinded, experienced echocardiographer using the standard techniques [18]. The echocardiographic parameters investigated included left ventricular diastolic diameter, left ventricular systolic diameter, and left atrial diameter from the parasternal long-axis view. Left ventricular ejection fraction was measured by the modified biplane Simpson’s method. Peak early (E) diastolic transmitral filling velocity and deceleration time of E diastolic transmitral filling velocity were measured.

### Statistical analysis

Data were analyzed using the Statistical Package for Social Sciences version 24 (SPSS Inc., Chicago, IL, USA). All quantitative data are expressed as mean ± SD, or median and interquartile range. The statistical significance of differences was analyzed using Student’s t-test for parametric continuous variables, and the Mann-Whitney U-test for nonparametric continuous variables. Categorical variables were compared using the Chi-square test or Fisher’s exact test. Correlations were analyzed using Spearman’s correlation analysis for variables. *P* < 0.05 was considered statistically significant.

## Results

### Baseline characteristics of the study groups

In the baseline characteristics, the stage C group had significantly higher levels of brain natriuretic peptide, larger left ventricular diameter and left atrial diameter, higher E diastolic transmitral filling velocity, and more frequent use of β-blocker, compared with the stage A or B group. The stage C group also had lower left ventricular ejection fraction, and lower levels of eGFR. The total ketone bodies in the blood, and the use of sodium-glucose linked transporter-2 inhibitor, which is associated with elevated blood ketone bodies, were not significantly different between the stage A or B group and stage C group (Table 1).

**Table 1:** Baseline characteristics of the study subjects

	Stage A or B (*n* = 20)	Stage C (*n* = 35)	*P* value
Age, years	69 ± 8	72 ± 20	0.431
Male	15 (75)	21 (60)	0.260
BMI, kg/m^2^	22 ± 4	21 ± 5	0.109
Smoking history	16 (80)	19 (54)	0.057
NYHA class, I/II/III/IV	–	3/28/4/0	–
SBP, mmHg	131 ± 19	124 ± 22	0.161
Heart rate, beats/min	71 ± 16	77 ± 19	0.077
Etiology			
Ischemic	–	9 (26)	–
Valvular	–	3 (9)	–
Cardiomyopathy	–	13 (37)	–
Others	–	10 (29)	–
Hypertension	13 (65)	26 (74)	0.466
COPD	4 (20)	2 (6)	0.119
Atrial fibrillation	3 (15)	11 (31)	0.178
Laboratory data			
eGFR, mL/min/1.73 m^2^	65 ± 18	44 ± 22	< 0.001
Total cholesterol, mg/dL	173 ± 32	163 ± 46	0.430
Total bilirubin, mg/dL	0.9 ± 0.3	0.9 ± 0.4	0.562
Glucose, mg/dL	125 ± 24	125 ± 37	0.403
Hemoglobin A1c, %	7.0 ± 1.2	6.7 ± 0.7	0.719
BNP, pg/mL (median, IQR)	32 (15–44)	274 (61–459)	< 0.001
TKB, μmL/L (median, IQR)	158 (68–271)	228 (75–448)	0.186
Echocardiography			
LVDd, mm	47 ± 7	52 ± 10	0.046
LVDs, mm	31 ± 7	40 ± 12	0.008
LVEF, %	58 ± 7	44 ± 16	0.005
LAD, mm	37 ± 6	44 ± 8	0.009
E, cm/s	0.6 ± 0.1	0.8 ± 0.3	0.032
Dct, msec	223 ± 54	198 ± 67	0.105
Medication			
ACE-I/ARB	10 (50)	22 (63)	0.352
β-blocker	7 (35)	24 (69)	0.016
Insulin	5 (25)	8 (23)	0.553
Oral antidiabetic agents except SGLT-2 inhibitor	12 (60)	16 (46)	0.308
SGLT-2 inhibitor	1 (5)	3 (9)	0.537

Values are mean ± SD or n (%), or median and IQR

*BMI* body mass index, *NYHA* New York Heart Association, *SBP* systolic blood pressure, *COPD* chronic obstructive pulmonary disease, *eGFR* estimated glomerular filtration rate, *BNP* brain natriuretic peptide, *IQR* interquartile range, *TKB* total ketone body, *LVDd* left ventricular diastolic diameter, *LVDs* left ventricular systolic diameter, *LVEF* left ventricular ejection fraction, *LAD* left atrial diameter, *E* peak early diastolic transmitral filling velocity, *Dct* deceleration time, *ACE-I* angiotensin-converting enzyme inhibitor, *ARB* angiotensin-receptor blocker, *SGLT* sodium-glucose linked transporter

### Exhaled acetone concentration between stage a or B group and stage C group

**Fig. 1:**
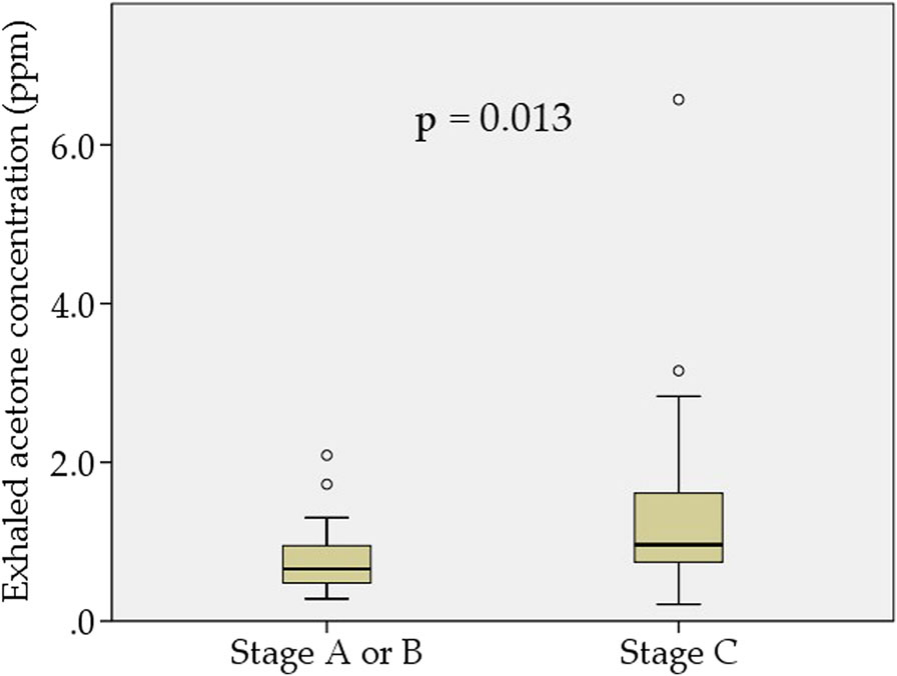
Exhaled acetone concentration between the stage A or B group and the stage C group. The stage C group had higher exhaled acetone concentration than the stage A or B group (*p* = 0.013)

The stage C group had higher exhaled acetone concentrations than those in the stage A or B group (*p* = 0.013, Fig. 1). The median exhaled acetone concentration (interquartile range) was 0.65 ppm (0.47–0.96 ppm) and 0.96 ppm (0.74–1.62 ppm) in the stage A or B group and the stage C group, respectively.

### Correlations of exhaled acetone concentration in all patients

**Fig. 2:**
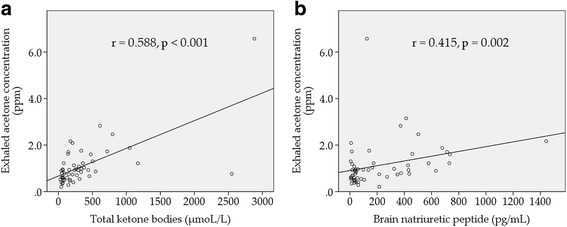
Correlations of exhaled acetone concentration with total ketone bodies (**a**) and brain natriuretic peptide (**b**). Exhaled acetone concentration was correlated with total ketone bodies (*r* = 0.588, *p* < 0.001) and brain natriuretic peptide (*r* = 0.415, *p* = 0.002)

**Fig. 3:**
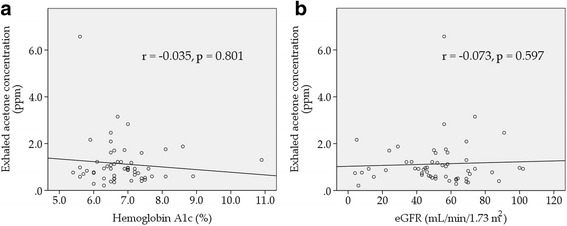
Correlations of exhaled acetone concentration with hemoglobin A1c (**a**) and eGFR (**b**). Exhaled acetone concentration was not correlated with hemoglobin A1c and estimated glomerular filtration rate (eGFR)

Exhaled acetone concentration was correlated with total ketone bodies (*r* = 0.588, *p* < 0.001, Fig. 2a) and brain natriuretic peptides (*r* = 0.415, *p* = 0.002, Fig. 2b). On the other hand, exhaled acetone concentration was not correlated with hemoglobin A1c (*r* = −0.035, *p* = 0.801, Fig. 3a) and eGFR (*r* = −0.073, *p* = 0.597, Fig. 3b).

## Discussion

To the best of our knowledge, this is the first study to reveal that diabetic patients with stage C heart failure have elevated exhaled acetone concentration, compared with diabetic patients with stage A or B heart failure. In addition, exhaled acetone concentration is correlated with heart failure severity in heart failure patients with diabetes mellitus.

Breath acetone concentration is reported to be a noninvasive biomarker of heart failure and is correlated with blood total ketone bodies, which are affected by diabetes mellitus [7, 8, 13, 19]. Many previous studies on breath acetone concentration and heart failure excluded the patients with diabetes mellitus in order to evaluate the direct relationship between breath acetone concentration and heart failure [7, 8, 14, 17]. However, diabetes mellitus is a common comorbidity in patients with heart failure [12]. Breath acetone concentration should have been evaluated in heart failure patients with diabetes mellitus to establish the usefulness of exhaled acetone concentration as a biomarker of heart failure. This study demonstrates that exhaled acetone concentration is elevated in stage C heart failure patients with diabetes mellitus, and could be a noninvasive biomarker in heart failure patients with diabetes mellitus.

The relationship between diabetes mellitus and exhaled acetone concentration has been controversial with some studies reporting elevated exhaled acetone concentrations in diabetes mellitus [19–22]. Other reports state that there are no obvious differences between diabetic patients and non-diabetic patients with regard to exhaled acetone concentrations [23]. In the present study, there was no correlation between exhaled acetone concentration and hemoglobin A1c in heart failure patients with diabetes mellitus. According to the present study, the severity of diabetes mellitus might not be directly correlated with exhaled acetone concentration in heart failure patients with diabetes mellitus.

Breath markers of kidney disease was described in a previous study [24]. Kidney disease did not cause elevated breath acetone concentration [24]. In our study, eGFR was not correlated with exhaled acetone concentration. Although the stage C group had both lower eGFR and elevated breath acetone concentration, the elevated acetone concentration was considered to be caused by heart failure.

Exhaled acetone concentration is affected by pulmonary congestion due to heart failure. We previously reported the correlation between exhaled acetone concentration and pulmonary capillary wedge pressure [17]. Increased pulmonary capillary hydrostatic pressure causes alveolar flooding and can increase the release of acetone into airways. Pulmonary congestive state itself might increase exhaled acetone concentration in stage C heart failure patients [17]. Exhaled acetone concentration could be a heart failure biomarker which reflects both blood ketone bodies and pulmonary congestion.

Among ketone bodies, acetone itself is correlated with elevated myocardial energy expenditure [5]. Elevated myocardial energy expenditure has been reported to be associated with reduced left ventricular ejection fraction and cardiovascular mortality [25]. Although the systemic and myocardial metabolism of ketone bodies remains unclear, detecting acetone might be a clue to understanding pathophysiological mechanisms in heart failure patients.

Measurement of exhaled acetone concentration is a noninvasive method, which is advantageous compared to the measurement of brain natriuretic peptide as it uses a needle for blood collection. Previous studies reported comparable diagnostic accuracy of exhaled acetone concentration with brain natriuretic peptide for heart failure in non-diabetic patients [8, 17].

A limitation of this study is that we used a small number of patients and a larger study population is required to evaluate exhaled acetone concentration as a biomarker of heart failure. Second, there were a lot of heart failure etiologies in the enrolled patients. The etiologies might have influenced the present results, and further study which evaluates the effects of exhaled acetone for heart failure etiologies with larger population is needed. Third, the correlation between exhaled acetone and blood total ketone bodies might be affected by other conditions. Exhaled acetone should be considered when diagnosing other conditions such as lung cancer, allergic asthma, and pulmonary congestion [9, 10, 17].

## Conclusion

Exhaled acetone concentration might be a noninvasive biomarker in heart failure patients with diabetes mellitus, correlating heart failure severity.

## Abbreviations

E: Peak early
eGFR: Estimated glomerular filtration rate
